# Psychometric Evaluation of the Persian Version of Adult Food Preferences Questionnaire (FPQ-Adult)

**DOI:** 10.1016/j.cdnut.2026.109399

**Published:** 2026-06-18

**Authors:** Zahra Heidari, Fahimeh Haghighatdoost, Shahnaz Amani Tirani, Awat Feizi

**Affiliations:** 1 Department of Biostatistics and Epidemiology, School of Health, Social Determinants of Health Research Center, Isfahan University of Medical Sciences, Isfahan, Iran; 2 Isfahan Cardiovascular Research Center, Cardiovascular Research Institute, Isfahan University of Medical Sciences, Isfahan, Iran; 3 Department of Community Nutrition, School of Nutrition and Food Sciences, Isfahan University of Medical Sciences, Isfahan, Iran; 4 Department of Biostatistics and Epidemiology, School of Health, Clinical Toxicology Research Center and Pharmaceutical Sciences Research Center, Isfahan University of Medical Sciences, Isfahan, Iran

**Keywords:** Food preferences, Adults, Validity, Reliability, Psychometrics

## Abstract

**Background:**

Preferences for consuming food items as determinants of food choices are significantly associated with diet- and health-related outcomes in adulthood.

**Objectives:**

The aim of the present study was to culturally adapt and investigate the psychometric properties of the Persian version of the food preferences questionnaire (FPQ-adult) among Iranian adults.

**Methods:**

This methodological cross-sectional study was conducted among 522 Persian-speaking adults, living in Isfahan, Iran. Translation of the FPQ was performed using forward–backward method. Intraclass correlation (ICC) and Cronbach α were used to assess test–retest reliability and internal consistency, respectively. Construct validity was investigated by using exploratory factor analysis (EFA). Known-group validity was assessed by using independent samples *t* test and analysis of variance. Convergent validity was determined using correlation analysis.

**Results:**

The internal and test–retest reliabilities for the FPQ-adult were in the range of moderate to excellent in all domains (intraclass correlation: 0.62–0.93; Cronbach α: 0.54–0.90). Construct validity led to extraction of 9 factors (fruit, vegetables, snacks, cereals-based dishes, rice and bread, processed dairy products and condiments, animal protein, fresh dairy products, and miscellaneous foods), explaining 41.73% of the total variance. The mean scores of fruits, vegetable, cereals-based dishes, and animal protein dimensions were significantly different between participants with elevated compared with low anxiety and depression scores, indicating good known-group validity. These 4 dimensions showed a significant positive correlation with age.

**Conclusions:**

The Persian version of the FPQ-adult demonstrated moderate to excellent test–retest reliability and acceptable to good internal consistency across all domains. The extracted factor structure supported construct validity, and significant differences in food preference dimensions between participants with elevated compared with low anxiety or depression scores confirmed known-group validity. These findings indicate that the Persian FPQ–adult has adequate psychometric properties for assessing food preferences in Persian-speaking community–dwelling adults.

## Introduction

Preferences for consuming food items are the evaluative attitudes that people express toward foods and include the qualitative evaluation of foods and the degree to which people like or dislike them [1]. Numerous factors, including environmental, physiological and neurophysiological, sensory acuity, psychological traits, and cultural and social aspects influence food choices and preferences [[2], [3], [4]]. Additionally, socioeconomic disparities and seasonal food availability play a crucial role in shaping dietary choices [5]. Dietary habits and preferences have changed in different parts of the world, such as Iran, with a dramatic increase in the consumption of high-fat foods such as fast food, sweetened beverages, and free sugar, whereas the consumption of fiber, fruits, and vegetables has decreased [6]. Obviously, these changes have led to an increase in risk factors for noncommunicable diseases, especially cardiovascular diseases, diabetes, obesity, and cancer [6,7].

Reliable measurement tools are essential for accurately assessing food choices and preferences, which serve as 1 of the important predictors of health outcomes [8]. Without culturally adapted instruments, research may provide inaccurate information about adults’ dietary preferences, potentially leading to ineffective policy recommendations and missed opportunities for targeted nutritional interventions, particularly in middle- and old-aged people [9]. Various questionnaires have been developed worldwide to evaluate food choice and preference. For example, both the original and modified food choice questionnaire (FCQ) [[10], [11], [12], [13], [14], [15], [16]] have been implemented, whereas the athlete FCQ has been used specifically for athletic population [17]. Additionally, several questionnaires have been designed to assess food preference in children and adolescence. Of particular significance is the questionnaire we validated in our previous study in a large sample of Iranian adolescents, which demonstrates strong cultural fit and psychometric soundness [[18], [19], [20], [21], [22], [23]]. Despite extensive global research on food preference assessment, studies developing and psychometrically validating such instruments specifically for Iranian adults remain scarce. The few investigations that exist such as the study by Jalali-Farahani and Amiri [24] reveal notable shortcomings. In their work, for instance, the validated FCQ primarily captures the factors influencing food choices (e.g., “It is important to me that the food I eat on a typical day keeps me healthy”) rather than directly evaluating specific food items. This narrow focus limits the instrument’s ability to offer detailed and practical insights into actual dietary behaviors, underscoring the need for a culturally adapted tool that comprehensively assesses food preferences [24].

Recognizing the distinctive dietary habits and behaviors, traditional culinary practices, and regional variations inherent to Persian-speaking populations further shaped by evolving socioeconomic influences and demographic diversity, this study emphasizes the urgent need for a culturally tailored instrument to accurately assess food preferences within Iranian communities. This study focuses on adapting and validating of the food preferences questionnaire (FPQ) for adults in Isfahan, Iran. This instrument was originally developed to evaluate food preferences among children and late adolescents (18–19 y) in the UK context [20,22], and its content has been expanded to incorporate a broader spectrum of foods items commonly consumed by Iranian adults. As stated, although we have previously developed a Persian version of this questionnaire for adolescents [6], evidence that the preferences for consuming different food items vary with various factors such as age, gender, and educational [16,25] underscore the necessity of examining its psychometric properties of this tool specifically in adult population. Therefore, we evaluated internal and test–retest reliability and different validity aspects of the extended FPQ-adult.

## Methods

### Study design and participants

This methodological cross-sectional study was conducted between May 2021 and June 2022 among 522 Persian-speaking adults aged 19 to 70 y in Isfahan, Iran, using a multistage cluster sampling of urban health centers, which serve defined geographic catchments and record citizen data in the Integrated Health System by randomly selecting 5 subcenters from each of the 2 main health networks (centers 1 and 2). To overcome the access limitations imposed by the COVID-19 pandemic, we used both electronic and printed questionnaire distribution methods: for the online survey, we randomly extracted mobile numbers for ∼1000 residents (∼100 per subcenter) from the Integrated Health System and sent them a Porsline-hosted link (https://survey.porsline.ir/n/survey/216184/build/) containing study objectives, consent information, and completion instructions, yielding 286 completed responses (28.6% response rate); concurrently, trained interviewers used convenience sampling at the same 10 subcenters to distribute 50 paper questionnaires each (500 total), of which 236 were completed on site. Eligible participants were Isfahan residents without major psychological, cognitive, or physical illnesses at recruitment. The combined sample of 522 respondents was then analyzed to assess the questionnaire’s construct validity, divergent validity, and known-group validity. This study complies with the Declaration of Helsinki and was performed according to ethics committee approval. The protocol of our study was ethically approved by the National Institute for Medical research Development (NIMAD; research project No: 982938, ethical approval No. IR.NIMAD.REC.1398.062).

### Procedures

#### The FPQ

Food preferences were assessed using the FPQ developed by Smith et al. [20]. The FPQ is a validated self-report tool originally designed for children and adolescents, adapted from a previously validated parent-proxy instrument [22]. The questionnaire comprises 62 food items, and respondents rate how much they like each item on a 5-point Likert scale: *1*) dislike a lot, *2*) dislike a little, *3*) neither like nor dislike, *4*) like a little, and *5*) like a lot. Respondents are instructed to rate only foods they have ever tried, regardless of current consumption. An additional option, “not applicable,” is available for foods they have never tried or do not remember trying [26].

The FPQ assesses preferences across 6 food categories: vegetables (18 items), fruit (7 items), meat/fish (12 items), dairy (10 items), snacks (9 items), and starches (6 items). A category-specific preference score is calculated by summing the item scores within each category and dividing by the number of items in that category.

The FPQ also asks whether respondents follow a specific eating plan (vegan, vegetarian, pescatarian, or none) and if they have allergies to some foods (e.g., peanuts). Reliability testing showed moderate to excellent test–retest intraclass correlations (ICCs: 0.61–0.95) and good internal consistency across subscales (Cronbach α = 0.77–0.89) [20,26].

For Iranian validation, culturally relevant items were incorporated to enhance local applicability, as detailed in our previous publication [6]. The psychometric properties of the Persian version of this questionnaire were previously evaluated in Iranian adolescents, and its factor structure was established in that population [6]. The internal and test–retest reliabilities for the Persian FPQ were in the range of good to excellent in all domains (Cronbach α: 0.76–0.96; ICC: 0.982–0.998). For the Persian version, 7 dimensions were extracted, including vegetables, fruit, dairy, snacks, meat/fish, starches, and miscellaneous foods [6]. In the current study, we assess the psychometric properties of the same Persian version of the FPQ in Iranian adults for the first time.

#### Translation and content validity

The details of the questionnaire translation process have been reported in our previous work [6]. Briefly, we got the initial developer’s permission at first and followed the method proposed by Beaton et al. [27] to translate the FPQ-62. In the forward stage, the questionnaire was translated by 2 fluent expert translators into Persian and then unified. After that, to compare with the original version, its final form was backward translated into English by 2 other translators based on conceptual balance. Finally, necessary modifications were made by the investigators, and the provisional Persian version of the FPQ questionnaire was prepared. The content validity of the prepared questionnaire was qualitatively evaluated by a team of dietitians. Some uncommon food items were removed or replaced with common counterparts in Iranian diet. Details of these changes have been reported in our previous work [6]. Finally, frequently used food items in Iran were added to complete the existing food items in original form. These changes led to an initial draft containing 93 food items for the Persian language version. However, in the current study, after merging some items, the final number was reduced to 89 items, which formed the basis for the analyses related to the evaluation of various types of validity and reliability.

### Psychometric analysis of the Persian FPQ

#### Construct validity

First, we tested the sample size adequacy and factorability by using Kaiser–Meyer–Olkin (values > 0.7) and Bartlett test of sphericity (*P* < 0.05) [28]. We then used the exploratory factor analysis (EFA) on 522 adults to determine the factor structure of the FPQ-adult. Principal component extraction approach and orthogonal Varimax rotation for interpretability were used. The number of factors was guided by eigenvalues >1 and scree plot. We kept items when their loading values were >0.20.

#### Known-groups validity

Known-groups validity was assessed based on the FPQ-adult ability to discriminate between different subgroups in terms of food preferences. For example, we hypothesized that the food preferences of a person with depression are significantly different from those of a healthy person. We compared the total score of the FPQ-adult and 9 nine dimensions between different groups based on sex, BMI, having allergy, anxiety, and depression. The validity of the measure is supported if mean of the FPQ-adult levels is significantly different between subgroups. The mean difference of scores of extracted food preference factors across categories of aforementioned variables was evaluated using independent Student *t* test and 1-way ANOVA. Normality of continuous data was evaluated using Kolmogorov–Smirnov test and Q–Q plot.

#### Convergent validity

Convergent validity was evaluated using Pearson correlation coefficient for normally distributed data or Spearman rank correlation coefficient for nonnormally distributed variables between the scores of each FPQ-adult subscale and external variables including age, BMI, and psychological symptoms (anxiety and depression) measured by the Hospital Anxiety and Depression Scale (HADS). The HADS consists of 14 items, with 7 items assessing anxiety (HADS-A) and 7 items assessing depression (HADS-D). We hypothesized that certain dietary preferences such as higher preference for fruits and vegetables would be negatively associated with psychological problems and that age and BMI would also show theoretically meaningful correlations with specific food preference dimensions. A validated Persian version of the HADS was used to determine depression and anxiety (Cronbach α: 0.78) [29]. It has a 4-point Likert scale ranging from 0 (not present) to 3 (considerable). The score for each subscale (HADS-A and HADS-D) ranged between 0 and 21 points (0–7, normal; 8–21, mild, moderate, or severe disorder). Based on the established cutoff scores for the HADS, participants with a score of ≥8 on HADS-A were classified as exhibiting symptoms of anxiety, and those with a score of ≥8 on HADS-D were classified as exhibiting symptoms of depression [29]. This cutoff point is widely used to identify possible cases of anxiety and depressive disorders in both clinical and community settings, offering an optimal balance of sensitivity and specificity.

#### Reliability

To assess test–retest reliability, the FPQ was administered twice to a subsample of participants with a 2-week interval. Considering the length of the questionnaire (89 items) and to minimize respondent burden while maintaining response accuracy, the retest questionnaire was distributed to 25 participants. Of these, 22 participants (88%) completed both administrations with adequate quality and were included in the test–retest analysis. This sample size is consistent with recommendations for test–retest reliability studies, where sample sizes of 20 to 30 participants are typically considered sufficient [30]. To evaluate test–retest reliability, the ICC with 95% CI using 2-way mixed model was estimated. The ICC values of <0.5 were regarded as poor, 0.5 to 0.75 as moderate, 0.75 to 0.9 as good, and >0.9 as excellent reliability [31]. Internal consistency was assessed using Cronbach α coefficient and the values of 0.70 to 0.8 and 0.8 to 0.9 were considered as acceptable and good, respectively, and >0.9 as excellent internal reliability [31].

#### Ceiling and floor effects

Floor and ceiling effects refer to the proportion of respondents who select the lowest (floor) or highest (ceiling) possible score on items or subscales of a questionnaire. These effects indicate limited sensitivity of the instrument in detecting variability at the lower or upper ends of the scale, potentially reducing its ability to distinguish between individuals with extreme levels of the measured construct. The presence of the ceiling and floor effects was determined by using the distribution of the lowest and highest possible FPQ-adult scores on a given subscale. The cutoff point of ≥15% was regarded as an indicator of floor and ceiling effects [31].

### Other measurements and statistical analysis

In this study, quantitative and categorical variables were expressed as mean ± SD and number (percentage), respectively. Missing data accounted for <2% of all variables and were handled using complete case analysis, meaning participants with missing data for a specific variable were excluded only from analyses involving that variable*.* A sensitivity analysis was also conducted to assess the potential impact of the administration mode (online compared with paper-and-pencil) on the psychometric properties of the Persian FPQ–adult. In all statistical analyses, *P* value of <0.05 was considered as significant level. All analyses were conducted using SPSS software (version 16; SPSS).

## Results

### Participant characteristics

A total of 522 adults [355 (68%) women[, participated in the current study. The mean ± SD age of participants was 37.72 ± 9.12 years. About 50% of the participants were classified as obese or overweight. Nearly 97% did not follow a specific dietary regimen. The prevalence of food allergy was estimated to be ∼18.6% and 15.7% among women and men, respectively. Based on the established cutoff score of ≥8 on the HADS, depressive symptoms (HADS-D) were identified in 12.1% of participants, and anxiety symptoms (HADS-A) were identified in 15.7% of participants (Table 1).

**TABLE 1:** Distribution of participant characteristics in total sample and by gender

Characteristic		Total (*N* = 522)	Female (*n* = 356)	Male (*n* = 166)
BMI category	Under weight	12 (2.3)	10 (2.8)	2 (1.2)
	Normal	246 (47.6)	182 (51.7)	63 (38.4)
	Over weight	204 (39.5)	125 (35.5)	79 (48.2)
	Obese	55 (10.6)	35 (9.9)	20 (12.2)
Education level	Under diploma (<12 y)	21 (4.0)	11 (3.1)	10 (6.1)
	Diploma (12 y)	76 (14.6)	38 (10.7)	38 (23.0)
	Collegiate (>12 y)	423 (81.3)	305 (86.2)	117 (70.9)
Marital status	Single	134 (26.1)	90 (25.8)	43 (26.2)
	Married	380 (73.9)	259 (74.2)	121 (73.8)
Anxiety (HADS-A)	Normal	327 (64.2)	213 (61.03)	114 (71.25)
	Borderline abnormal	102 (20.0)	75 (21.49)	27 (16.88)
	Abnormal	80 (15.7)	61 (17.48)	19 (11.88)
Depression (HADS-D)	Normal	340 (67.6)	226 (65.32)	113 (72.44)
	Borderline abnormal	102 (20.3)	73 (21.10)	29 (18.59)
	Abnormal	61 (12.1)	47 (13.58)	14 (8.97)
Do you identify as any of the following?	No special diet	501 (96.5)	343 (96.6)	157 (94.6)
	Vegan	2 (0.4)	1 (0.3)	1 (0.6)
	Vegetarian	7 (1.3)	5 (1.4)	2 (1.2)
	Pescetarian (no meat, but eat fish and/or shellfish)	9 (1.7)	4 (1.1)	5 (3.0)
Are you allergic to any of the following food items?	No allergies to specific food items	430 (82.4)	289 (81.4)	140 (84.3)
	Peanuts (yes)	12 (2.30)	8 (2.25)	4 (2.41)
	Tree nuts (yes)	7 (1.34)	6 (1.69)	1 (0.60)
	Sesame (yes)	3 (0.57)	1 (0.28)	2 (1.20)
	Dairy (yes)	8 (1.53)	7 (1.97)	1 (0.60)
	Shellfish (yes)	9 (1.72)	7 (1.97)	2 (1.20)
	Fish (yes)	6 (1.15)	4 (1.13)	2 (1.20)
	Egg (yes)	8 (1.53)	5 (1.41)	3 (1.81)
	Wheat/gluten (yes)	1 (0.19)	2 (0.56)	1 (0.60)
	Soya (yes)	4 (0.77)	3 (0.85)	2 (1.20)
	Celery (yes)	4 (0.77)	6 (1.69)	1 (0.60)
	Mustard (yes)	10 (1.92)	40 (11.27)	4 (2.41)
	Eggplant (yes)	56 (10.73)	19 (5.35)	16 (9.64)
	Tomato (yes)	24 (4.60)	8 (2.25)	5 (3.01)
Age (y)		37.72 ± 9.12	37.83 ±8.76	37.52 ±9.88
BMI (kg/m^2^)		25.11 ± 3.87	24.75 ±3.98	25.88 ±3.52
Anxiety (HADS-A)		6.52 ± 4.19	6.78 ±4.28	5.95 ±3.94
Depression (HADS-D)		6.14 ± 3.61	6.43 ±3.66	5.51 ±3.43

Values are *n* (%) for categorical or mean ± SD for continuous variables. The discrepancy between the number of participants across categories of some variables and the total sample size is due to missing data in those variables.

Abbreviation: HADS, Hospital Anxiety and Depression Scale.

### Construct validity

Construct validity was evaluated by using EFA. We identified 9 dimensions from Persian FPQ–adult measure based on 89 food items: *1*) a fruit factor, which characterized by high interest in peaches, apricots, grapes, cantaloups, sour cherries, pomegranates, oranges, yellow plums, cherries, tangerines, honeydew melons, watermelons, sour green plums, melons, apples, figs, kiwis, mulberries, sweet lemons, strawberries, and persimmons; *2*) a vegetable factor, was defined by high liking for green peppers, bell peppers, celery, onions, peas, raw tomatoes, salad leaves (e.g., lettuce), red peppers, green beans, turnips, garlic, beetroots, cabbage verities (including cauliflower and brussels sprouts), spinach, broccoli, carrots, cucumber, mushrooms, tomato paste and corn; *3*) a snack food factor, which characterized by high interest in chocolate biscuits, cake, chocolate, ice cream, salty snacks (such as puffs, chips, and crisps), plain biscuits, chewable jelly chocolates (such as pastilles and jelly dragees), sausages, ham, ketchup, and various flavored milk (such as cocoa milk, banana milk, carrot milk, and coconut milk); *4*) a cereals-based dish factor, which is characterized by high interest in Persian rice pudding (shir berenj), haleem, porridge, baked legumes (lentils, beans, chickpeas, mung beans, and cobs), corn, eggs (boiled, mashed, or fried), potatoes (boiled or mashed), cheese, and yogurt; *5*) a rice and bread factor, which characterized by high interest in white baguette breads without bran (such as hamburger bread), wholemeal baguette, traditional whole meal breads (such as Sangak), refined flatbreads (such as Lavash, Tufton, and Barbari), rice and beans, plain boiled rice, French fries, and breakfast cereal; *6*) processed dairy product and condiment factor, which characterized by strong interest in cheese, other cheeses (such as Parmesan cheese, mozzarella, blue cheese, Camembert, and Gouda), cream, cream cheese (such as mascarpone cheese), mayonnaise, butter, margarine, and ketchup; *7*) an animal protein factor, which characterized by high interest in fatty fish, low-fat fish, white meat burger (chicken or fish), lamb, chicken, beef burgers, beef, and smoked salmon; *8*) a fresh dairy product factor, which characterized by high interest in plain low-fat milk, plain full fat milk, custard, flavored milk (such as cocoa, banana, carrot, and coconut) and yogurt; *9*) a miscellaneous food factor, which characterized by high preference in avocadoes, parsnip, smoked salmon, bran cereals (such as wheat or rice bran), and grapefruit. These factors were accounted for 8.0%, 7.59%, 5.33%, 4.04%, 3.81%, 3.48%, 3.34%, 3.26%, and 2.88% of total variance, respectively. A Kaiser–Meyer–Olkin value of 0.768 and *P* of < 0.05 for the Bartlett test confirmed data viability for conducting a reliable factor analysis in terms of sample size adequacy and factorability.

Table 2 provides the factor loadings of 9 extracted factors from FPQ-adult items. During the construct validity assessment, the lentil soup and tuna fish items was removed due to their low factor loadings. We also combined various yogurts and 2 cheese varieties (i.e., Iranian white cheese and traditional cheese) to improve interpretability. In addition, the chickpea item was merged with the baked legumes item.

**TABLE 2:** Factor loadings of food items on the 9 subscales of the Persian FPQ-Adult derived from exploratory factor analysis for evaluating constructed validity

Food items	FPQ—adult’s subscales								
	Fruits	Vegetables	Snacks	Cereals- based dishes	Rice and bread	Processed dairy products and condiments	Animal protein	Fresh dairy products	Miscellaneous foods
Peaches	0.605	—	—	—	—	—	—	—	—
Apricots	0.603	—	—	—	—	—	—	—	—
Grape	0.599	—	—	—	—	—	—	—	—
Cantaloup	0.592	—	—	—	—	—	—	—	—
Sour cherry	0.573	—	—	—	—	—	—	—	—
Pomegranate	0.558	—	—	—	—	—	—	—	—
Oranges	0.557	—	—	—	—	—	—	—	—
Yellow plum	0.555	—	—	—	—	—	—	—	—
Cherry	0.547	—	—	—	—	—	—	—	—
Tangerine	0.528	—	—	—	—	—	—	—	—
Dew melon	0.526	—	—	—	—	—	—	—	—
Watermelon	0.520	—	—	—	—	—	—	—	—
Sour green plum	0.505	—	—	—	—	—	—	—	—
Melon	0.489	—	—	—	—	—	—	—	—
Apples	0.485	—	—	—	—	—	—	—	—
Fig	0.485	—	—	—	—	—	—	—	—
Kiwi	0.457	—	—	—	—	—	—	—	—
Mulberry	0.456	—	—	—	—	—	—	—	—
Sweet lemon	0.415	—	—	—	—	—	—	—	—
Strawberries	0.404	—	—	—	—	—	—	—	—
Persimmon	0.327	—	—	—	—	—	—	—	—
Green pepper	—	0.641	—	—	—	—	—	—	—
Bell pepper	—	0.627	—	—	—	—	—	—	—
Celery	—	0.594	—	—	—	—	—	—	—
Onion	—	0.592	—	—	—	—	—	—	—
Peas	—	0.571	—	—	—	—	—	—	—
Raw tomatoes	—	0.555	—	—	—	—	—	—	—
Salad leaves (e.g., lettuce)	—	0.546	—	—	—	—	—	—	—
Red peppers	—	0.541	—	—	—	—	—	—	—
Green beans	—	0.538	—	—	—	—	—	—	—
Turnip	—	0.528	—	—	—	—	—	—	—
Garlic	—	0.527	—	—	—	—	—	—	—
Beetroot		0.511	—	—	—	—	—	—	—
Cabbage (e.g., cabbage, cauliflower, and Brussels sprouts)	—	0.504	—	—	—	—	—	—	—
Spinach	—	0.488	—	—	—	—	—	—	—
Broccoli	—	0.476	—	—	—	—	—	—	—
Carrots	—	0.476	—	—	—	—	—	—	—
Cucumber	—	0.454	—	—	—	—	—	—	—
Mushrooms	—	0.453	—	—	—	—	—	—	—
Tomato paste	—	0.412	—	—	—	—	—	—	—
Chocolate biscuits	—	—	0.736	—	—	—	—	—	—
Cake	—	—	0.726	—	—	—	—	—	—
Chocolate	—	—	0.690	—	—	—	—	—	—
Ice cream	—	—	0.611	—	—	—	—	—	—
Salty snacks (e.g., puffs, chips, and crisps)	—	—	0.586	—	—	—	—	—	—
Plain biscuits	—	—	0.574	—	—	—	—	—	—
Chewable jelly chocolates (eg pastilles and jelly dragees)	—	—	0.512	—	—	—	—	—	—
Sausages	—	—	0.369	—	—	—	—	—	—
Ham	—	—	0.347	—	—	—	—	—	—
Rice pudding (rice milk)	—	—	—	0.542	—	—	—	—	—
Haleem	—	—	—	0.533	—	—	—	—	—
Porridge	—	—	—	0.505	—	—	—	—	—
Baked legumes (e.g., lentils, beans, chickpeas, mung beans, and cobs)	—	—	—	0.404	—	—	—	—	—
Corn	—	0.372	—	0.380	—	—	—	—	—
Eggs (boiled, scrambled, or fried)	—	—	—	0.354	—	—	—	—	—
Potatoes (boiled or mashed)	—	—	—	0.324	—	—	—	—	—
Cheese	—	—	—	0.299	—	0.232	—	—	—
Baguette bread without bran (e.g., hamburger bread)	—	—	—	—	0.594	—	—	—	—
Wholemeal baguette bread	—	—	—	—	0.567	—	—	—	—
Traditional wholemeal bread (like.g., Sangak)	—	—	—	—	0.547	—	—	—	—
Traditional bread without bran (e.g., lavash bread, Tufton bread, and Berberi bread)	—	—	—	—	0.528	—	—	—	—
Rice and beans	—	—	—	—	0.523	—	—	—	—
Plain boiled rice	—	—	—	—	0.516	—	—	—	—
French fries	—	—	—	—	0.452	—	—	—	—
Breakfast cereal	—	—	—	—	0.373	—	—	—	—
Other cheeses (e.g., Parmesan cheese, mozzarella, blue cheese, Camembert, and Gouda)	—	—	—	—	—	0.519	—	—	—
Cream	—	—	—	—	—	0.512	—	—	—
Cream cheese (e.g., mascarpone cheese)	—	—	—	—	—	0.510	—	—	—
Mayonnaise	—	—	—	—	—	0.505	—	—	—
Butter	—	—	—	—	—	0.497	—	—	—
Margarine	—	—	—	—	—	0.475	—	—	—
Ketchup	—	—	0.317	—	—	0.449	—	—	—
Fatty fish (e.g., salmon, salmon, sardines, and mackerel)	—	—	—	—	—	—	0.636	—	—
Low-fat fish (e.g., kilka fish, milk fish, shourideh, halva, serkhu, and tilapia)	—	—	—	—	—	—	0.602	—	—
White meat burger (chicken or fish)	—	—	—	—	—	—	0.561	—	—
Lamb	—	—	—	—	—	—	0.554	—	—
Chicken	—	—	—	—	—	—	0.494	—	—
Beef burgers	—	—	—	—	—	—	0.489	—	—
Beef	—	—	—	—	—	—	0.402	—	—
Plain low-fat milk	—	—	—	—	—	—	—	0.784	—
Plain full fat milk	—	—	—	—	—	—	—	0.772	—
Custard	—	—	—	—	—	—	—	0.449	—
Other types of milk (e.g., cocoa milk, banana milk, carrot milk, and coconut milk)	—	—	0.398	—	—	—	—	0.441	—
Yogurt	—	—	—	0.304	—	—	—	0.336	—
Avocadoes	—	—	—	—	—	—	—	—	0.614
Parsnip	—	—	—	—	—	—	—	—	0.476
Smoked salmon	—	—	—	—	—	—	0.383	—	0.444
Bran cereal (such as wheat bran or rice bran)	—	—	—	—	—	—	—	—	0.423
Grapefruit	—	—	—	—	—	—	—	—	0.403
Variance explained^1^ (%)	8.00	7.59	5.33	4.04	3.81	3.48	3.34	3.26	2.88

Abbreviations: FPQ, food preferences questionnaire; HADS, Hospital Anxiety and Depression Scale.

1 Exploratory factor analysis with principal component extraction method and Varimax rotation on *n* = 522. factor loadings of <0.2 are not shown for simplicity.

For the food items examined in the questionnaire, whose corresponding factors were presented in Table 3 based on the factor analysis, the frequency of “not applicable” responses were assessed. This response option indicates that participants had never tried or did not recognize the food item.

**TABLE 3:** Comparison of the subscales score of FPQ–adult questionnaire between different categories of participants’ characteristics (known-group validity)

Characteristic		Fruit	Vegetables	Snacks	Cereals-based dishes	Rice and bread	Processed dairy products and condiments	Animal protein	Fresh dairy products	Miscellaneous foods
Gender	Female (*n* = 356)	94.94 ± 9.46	83.44 ± 10.94	43.90 ± 7.07	37.00 ± 5.00	34.83 ± 4.09	26.51 ± 5.81	33.02 ± 5.10	20.55 ± 3.86	20.62 ± 4.22
	Male (*n* = 166)	95.90 ± 9.12	80.42 ± 13.42	42.27 ± 8.43	36.70 ± 5.19	33.18 ± 5.27	26.75 ± 5.85	33.33 ± 5.20	20.40 ± 3.85	20.39 ± 4.31
	*P*^1^	0.277	0.007	0.022	0.527	<0.001	0.654	0.519	0.664	0.558
BMI	Under weight (*n* = 12)	95.25 ± 7.33	78.17 ± 11.82	44.58 ± 7.69	36.54 ± 5.56	36.58 ± 4.17	25.92 ± 8.27	34.08 ± 3.60	19.17 ± 4.93	20.83 ± 3.51
	Normal (*n* = 246)	95.50 ± 9.41	82.83 ± 11.79	43.90 ± 7.23	37.02 ± 5.05	34.44 ± 4.18	26.94 ± 5.79	33.35 ± 5.28	20.72 ± 3.81	20.48 ± 4.29
	Over weight (*n* = 204)	95.47 ± 9.45	82.79 ± 12.29	43.08 ± 7.94	37.16 ± 5.07	34.24 ± 5.06	26.48 ± 5.74	33.05 ± 4.89	20.43 ± 3.74	20.80 ± 4.20
	Obese (*n* = 55)	93.80 ± 7.86	81.18 ± 10.57	42.29 ± 7.16	35.69 ± 4.89	33.27 ± 4.20	25.73 ± 5.55	32.62 ± 4.62	20.35 ± 4.01	19.98 ± 4.19
	*P*^1^	0.65	0.461	0.401	0.275	0.108	0.503	0.687	0.5	0.608
Allergy	Yes (*n* = 92)	92.80 ± 10.17	80.52 ± 13.21	43.50 ± 7.73	36.32 ± 5.57	33.60 ± 5.08	26.20 ± 6.29	32.58 ± 5.69	19.92 ± 4.06	20.95 ± 3.99
	No (*n* = 430)	95.77 ± 9.09	82.90 ± 11.50	43.35 ± 7.53	37.02 ± 4.94	34.45 ± 4.43	26.67 ± 5.71	33.24 ± 4.99	20.63 ± 3.79	20.45 ± 4.30
	*P*^1^	0.006	0.081	0.86	0.228	0.103	0.483	0.262	0.112	0.313
Anxiety (HADS-A)	Normal (*n* = 327)	95.99 ± 8.34	83.00 ± 11.45	43.16 ± 7.70	37.41 ± 4.70	34.35 ± 4.33	26.53 ± 5.74	33.53 ± 4.73	20.71 ± 3.65	20.76 ± 4.23
	Borderline abnormal (*n* = 102)	95.11 ± 9.55	83.49 ± 11.59	43.86 ± 7.05	36.93 ± 5.47	34.12 ± 4.70	26.82 ± 6.39	32.87 ± 4.94	20.53 ± 4.10	20.66 ± 3.82
	Abnormal (*n* = 80)	92.15 ± 12.12	79.18 ± 13.64	43.55 ± 7.69	34.85 ± 5.60	34.55 ± 5.06	26.25 ± 5.27	31.68 ± 6.59	19.62 ± 4.33	19.46 ± 4.57
	*P*^1^	0.004	0.023	0.692	<0.001	0.811	0.801	0.013	0.076	0.045
Depression (HADS-D)	Normal (*n* = 340)	96.36 ± 7.98	83.17 ± 11.42	43.61 ± 7.56	37.56 ± 4.71	34.30 ± 4.46	26.71 ± 5.93	33.53 ± 4.72	20.81 ± 3.73	20.71 ± 4.13
	Borderline abnormal (*n* = 102)	94.25 ± 10.66	82.15 ± 11.61	42.26 ± 7.77	36.12 ± 5.36	34.11 ± 4.78	26.26 ± 5.75	32.75 ± 5.08	19.56 ± 3.84	19.89 ± 4.06
	Abnormal (*n* = 61)	90.72 ± 12.22	79.61 ± 12.31	43.41 ± 6.56	34.71 ± 5.55	34.64 ± 4.52	26.33 ± 5.07	31.95 ± 6.05	20.22 ± 3.99	20.25 ± 4.97
	*P*^1^	<0.001	0.081	0.283	<0.001	0.769	0.747	0.045	0.012	0.205

Values are mean ± SD. The discrepancy between the number of participants across categories of some variables and the total sample size is due to missing data in those variables.

Abbreviations: FPQ, food preferences questionnaire; HADS, Hospital Anxiety and Depression Scale.

1 *P* value resulted from independent samples *t* test or ANOVA and P<0.05 is statistically significant.

The highest prevalence of “not applicable” responses was observed for avocado (39.8%), followed by margarine (29.1%), custard (27.4%), and smoked fish (23.0%). Other items with relatively high “not applicable” rates included other cheeses (12.8%), grapefruit (11.7%), and breakfast cereals (9.4%). In contrast, items such as eggs, plain rice, chicken, lamb, and most fruits (e.g., grapes, apples, and pomegranates) had very low rates of “not applicable” responses (<1%), indicating high familiarity and consumption experience among participants.

### Known-groups validity

The results of known-group validity are presented in Table 3. For known-groups validity evaluation, based on gender, women scored markedly higher than men on the vegetables, snacks, and rice and bread subscales, whereas no gender differences emerged for fruit, cereals-based dishes, processed dairy products and condiments, animal protein, fresh dairy products, or miscellaneous foods. Across BMI categories (underweight, normal, overweight, and obese), no significant differences were observed on any of the 9 subscales, indicating that FPQ-adult scores are independent of BMI. Participants with a self-reported food allergy had significantly lower fruit scores than those without allergy, with no allergy-related differences on vegetables, snack foods, cereals-based dishes, rice and bread, processed dairy products and condiments, animal protein, fresh dairy products, or miscellaneous foods. Regarding anxiety, participants with elevated anxiety scores (HADS-A ≥8) had significantly lower mean preferences than those with low anxiety for fruit (*P* = 0.004), vegetables (*P* = 0.023), cereals-based dishes (*P* < 0.001), animal protein (*P* = 0.013), and miscellaneous foods (*P* = 0.045). No significant differences were observed between the groups for snacks, rice and bread, processed dairy products and condiments, or fresh dairy products.

Regarding depression, participants with elevated depression scores (HADS-D ≥8) had significantly lower mean preferences than those with low depression for fruit (*P* < 0.001), cereals-based dishes (*P* < 0.001), animal protein (*P* = 0.045), and fresh dairy products (*P* = 0.012). No significant differences were observed between the groups for vegetables, snacks, rice and bread, processed dairy products and condiments, or miscellaneous foods (Table 3).

### Convergent validity

Convergent validity was supported by significant negative correlations between 5 FPQ-adult subscales (ie fruit, vegetables, cereals-based dishes, animal protein, and fresh dairy products) and psychological distress measure (*P* < 0.05). The results also showed that with increasing age, interest in consuming snack foods, rice and bread, and processed dairy products and condiments significantly decreased while the desire to consume fruit, vegetables, cereals-based dishes, and animal protein increased (*P* < 0.05) (Table 4).

**TABLE 4:** Correlations of the subscales score of FPQ–adult questionnaire with age, BMI, and psychological problems to assess the convergent validity

FPQ–adult questionnaire’s subscales	Age	BMI	Anxiety (HADS-A)	Depression (HADS-D)
Fruit	0.120∗∗	−0.049	−0.125∗∗	−0.181∗∗
Vegetables	0.175∗∗	−0.020	−0.099∗	−0.116∗∗
Snacks	−0.162∗∗	−0.044	0.028	−0.015
Cereals- based dishes	0.092∗	−0.036	−0.156∗∗	−0.204∗∗
Rice and bread	−0.133∗∗	−0.094∗	−0.003	0.005
Processed dairy products and condiments	−0.136∗∗	−0.047	−0.003	−0.021
Animal protein	0.089∗	−0.056	−0.127∗∗	−0.120∗∗
Fresh dairy products	−0.039	−0.018	−0.102∗	−0.098∗
Miscellaneous foods	−0.035	−0.042	−0.106∗	−0.067
Total score	0.033	−0.067	−0.121∗∗	−0.153∗∗

Correlation resulted from Pearson correlation coefficient for normally and Spearman rank correlation coefficient for nonnormally distributed variables.

Abbreviations: FPQ, food preferences questionnaire; HADS, Hospital Anxiety and Depression Scale.

∗ *Significant at P* < 0.05.

∗∗ *Significant at P* < 0.01 level.

### Reliability analyses

The reliability analysis results and descriptive statistics for the 9 FPQ-adult scales are shown in Table 5. The ICC coefficient for the total score of the FPQ-adult suggests good test–retest reliability (ICC: 0.874; 95% CI: 0.681, 0.950; *P* < 0.001). The ICC coefficients for the extracted subscales including fruit, vegetables, snacks, cereals-based dishes, and processed dairy products and condiments were estimated to be >0.8 indicating good test–retest reliability. However, the ICC coefficients for the other subscales including rice and bread, animal protein, fresh dairy products, and miscellaneous foods indicated moderate test–retest reliability.

**TABLE 5:** Descriptive statistics, reliability coefficients (Cronbach α and test–retest ICC), and floor/ceiling effects for the total FPQ-Adult and its 9 subscales

Subscale	Mean ±SD	Cronbach α	ICC (95% CI)	Floor (%)	Ceiling (%)
Fruit	95.25 ± 9.35	0.882	0.877 (0.726, 0.947)	1 (0.2)	1 (0.2)
Vegetables	82.48 ± 11.85	0.885	0.926 (0.831, 0.969)	1 (0.2)	3 (0.6)
Snacks	43.37 ± 7.56	0.788	0.843 (0.653, 0.933)	2 (0.4)	2 (0.4)
Cereals- based dishes	36.90 ± 5.06	0.683	0.862 (0.668, 0.943)	1 (0.2)	1 (0.2)
Rice and bread	34.30 ± 4.56	0.720	0.686 (0.244, 0.870)	1 (0.2)	2 (0.4)
Processed dairy products and condiments	26.58 ± 5.81	0.717	0.837 (0.599, 0.934)	1 (0.2)	1 (0.2)
Animal protein	33.12 ± 5.12	0.676	0.644 (0.143, 0.852)	1 (0.2)	1 (0.2)
Fresh dairy products	20.50 ± 3.85	0.715	0.622 (0.089, 0.843)	1 (0.2)	1 (0.2)
Miscellaneous foods	20.54 ± 4.25	0.540	0.647 (0.150, 0.854)	1 (0.2)	6 (1.1)
Total score	372.82 ± 34.17	0.900	0.874 (0.681, 0.950)	1 (0.2)	1 (0.2)

All presented data are based on *N* = 522, except for ICC, which was obtained from a subsample of *n* = 22.

Abbreviations: FPQ, food preferences questionnaire; HADS, Hospital Anxiety and Depression Scale; ICC, intraclass correlation coefficient.

Cronbach α coefficients indicating item internal consistency for each subscale are presented in Table 5. Most subscales demonstrated satisfactory internal consistency ranging from 0.715 to 0.885. The overall FPQ-adult total score achieved a Cronbach α coefficient of 0.90, suggesting excellent internal consistency. However, internal consistency was moderate for cereals-based dishes, animal protein, and miscellaneous foods dimensions.

### Ceiling and floor effect

The percentage of respondents scoring at the highest level (i.e., ceiling effect) was between 0.2% and 1.1% for all subscales, whereas the percentage of participants scoring at the lowest level, i.e., <0.5% (floor effect) was minimal for all subscales. These negligible effects confirm that the questionnaire retains full sensitivity and discriminative capacity across its entire scoring spectrum (Table 5).

### Sensitivity analysis results

A sensitivity analysis was conducted to assess the potential impact of the administration mode (online compared with paper-and-pencil) on the psychometric properties of the Persian FPQ–adult. The results are presented in Supplemental Tables 1 to 3. The internal consistency for each factor was calculated separately for the online (*n* = 286) and paper (*n* = 236) subgroups. The results demonstrated high stability across both formats, with Cronbach α values for the online and paper groups being 0.617 and 0.713 for animal protein, 0.670 and 0.692 for rice and bread, 0.696 and 0.725 for cereals-based dishes, 0.874 and 0.890 for fruit, 0.865 and 0.900 for vegetables, 0.797 and 0.780 for snacks, 0.704 and 0.730 for processed dairy, 0.700 and 0.734 for fresh dairy, and 0.487 and 0.574 for miscellaneous foods, respectively. The overall reliability of the questionnaire was excellent in both administration formats, with Cronbach α coefficients of 0.902 for the online group and 0.914 for the paper group. The consistency of these reliability estimates between the 2 modes confirms that the questionnaire’s validity and reliability are robust and not influenced by the method of administration, supporting the integrity of pooling data from both sources for the main analysis (Supplemental Table 1).

To further assess the robustness of our findings, we examined the convergent validity of the FPQ-adult factors by analyzing their correlations with age, BMI, anxiety, and depression separately for the online and paper-based groups. The pattern of correlations was largely consistent between the 2 subsamples and aligned with the results reported for the whole sample. For instance, fruit and vegetable consumption showed significant positive correlations with age in the online group (*r* = 0.156; *P* < 0.01; *r* = 0.228; *P* < 0.01) and similar positive trends in the paper group, although the latter did not reach statistical significance. The significant negative associations between healthy food factors (fruit, vegetables, animal protein) and psychological distress (anxiety and depression) observed in the full sample were also replicated in the online subsample. Conversely, the significant negative correlation between the rice and bread factor and BMI, observed in the full sample, was specifically evident in the paper subsample (*r* = −0.137; *P* < 0.05). Overall, the similar pattern and magnitude of correlations across the 2 administration modes confirm that the convergent validity of the Persian FPQ–adult is stable and not dependent on the method of questionnaire completion, supporting the pooled analysis (Supplemental Table 2).

We also evaluated the robustness of the known-groups validity of the Persian FPQ–adult, by conducting stratified analyses in terms of questionnaire administration mode (online compared with paper). The pattern of differences across subgroups (gender, BMI categories, allergy status, anxiety, and depression categories) was generally consistent between the 2 formats and aligned with the findings from the whole sample. For instance, the significant gender differences observed in the whole sample for vegetables and snacks were replicated in the paper group (vegetables: *P* = 0.001; snacks: *P* = 0.004), whereas the online group showed similar directional trends. The association between allergy status and fruit consumption, significant in the whole sample (*P* = 0.006), was specifically evident in the online group (*P* = 0.009). Regarding psychological distress, the significant inverse associations between anxiety/depression and healthy food factors (fruit, vegetables, and animal protein) observed in the whole sample were predominantly driven by the online subsample, whereas the paper group demonstrated similar patterns but with fewer statistically significant differences, likely due to smaller sample sizes. Overall, these stratified analyses confirm that the known-groups validity of the Persian FPQ–adult is stable across different administration formats, supporting the robustness of the instrument (Supplemental Table 3).

Due to the inadequate sample size in each subgroup (online: *n* = 286; paper: *n* = 236) relative to the large number of questionnaire items, factor analysis could not be reliably performed for each administration mode separately, as this would have yielded unstable and potentially misleading results. Therefore, this aspect of validity was not examined in the stratified analysis.

## Discussion

In the current study, we evaluated the psychometric properties of the Persian version of FPQ-adult. FPQ-adult, one of the few versions of fully validated questionnaires for assessing adults’ preferences for consuming foods. EFA supported 9-factor structure (fruit, vegetables, snack foods, cereals-based dishes, rice and bread, processed dairy products and condiments, animal protein, fresh dairy products, and miscellaneous foods). The Persian FPQ–adult demonstrated good to acceptable test–retest reliability and internal consistency. Healthy adults had higher scores of fruit, vegetable, cereals-based dishes and animal protein dimensions than depressed and anxious people, indicating good known-group validity. The convergent validity was also satisfactory.

The results of Cronbach α and ICC coefficients in our adult sample indicate that the FPQ-adult maintains acceptable internal consistency and test–retest reliability, largely mirroring the robustness of the original questionnaire (original α: vegetables, 0.89; fruit, 0.84; meat/fish, 0.81; dairy, 0.77; snacks, 0.80; original ICCs: 0.61–0.95) [20]. In our data, total score reliability was strong (α: 0.900; ICC: 0.874), and subscale α’s ranged from 0.540 to 0.885, with ICCs from 0.622 to 0.926 all exceeded acceptable thresholds. The animal protein subscale showed lower internal consistency than originally reported (α: 0.676 compared with 0.81) and a modest ICC (0.644), whereas the miscellaneous foods subscale exhibited the weakest reliability (α: 0.540; ICC: 0.647). By comparison, our previous adolescent validation demonstrated higher internal consistency (α: 0.76–0.96) and markedly stronger test–retest reliability (ICCs: 0.982–0.998) [6]. The differences in Cronbach α and ICC between our validation and the original FPQ can largely be traced to the expanded item pools in our subscales scale length directly influences α, since adding more items with even modest interitem correlations can boost internal consistency, while also introducing heterogeneity that may counteract that effect. Likewise, ICC estimates depend on both true score variance and measurement error: a larger, more diverse item set not only can amplify variability but also increases the opportunity for random error, thereby altering reliability coefficients. Beyond item count, variations in sample characteristics (age range and cultural food practices), translation and adaptation nuances, the length of the test–retest interval, and administration conditions (eg self-report compared with supervised completion) affect consistency over time and interitem agreement, helping to explain why certain subscales in our study diverge from the original reliability benchmarks.

Although the original English FPQ did not undergo a formal EFA, our 9-factor structure closely parallels those reported by Smith et al. [20] in a large adolescent sample (n = 2865 twins aged 18-19 y) and Wardle et al. [21]. The study of 4-y-old UK children (*n* = 214) by Wardle et al. [22], using principal axis factoring with Oblimin rotation, produced 4 factors including vegetables, desserts, meat and fish, and fruits, explaining 24% of variance. In our previous work on Iranian adolescents (*n* = 452, ages 12–18 y), principal axis factoring with Varimax rotation identified 7 factors including vegetables, fruits, dairy, snacks, meat/fish, starches, and miscellaneous foods, which explained 37.8% of variance [6]. Earlier instruments such as the FCQ developed by Steptoe et al. [15], food preference inventory from Wardle et al. [32], and Leeds FPQ [33] also identify core domains, i.e., fruits, vegetables, dairy, and proteins, but vary in item coverage, response scales, and administration mode. By distinguishing rice and bread from cereal-based dishes, the Persian FPQ–adult uniquely captures the central role of staple grains in the Iranian diet. Discrepancies across studies likely reflect differences in sample size and composition, number and wording of items, extraction method (principal axis compared with principal components), rotation technique (orthogonal compared with oblique), age range, socioeconomic and cultural context, and participants’ ethnic backgrounds.

We examined the known-group validity based on various factors including sex, allergy, BMI, and mental health. The mean scores of vegetables, snacks, and bread/rice factors were significantly higher in women than in men. Conversely, in Iranian adolescents, boys had higher mean scores for these dimensions than girls [6]. In the study by Caine-Bish and Scheule [23], fruits and vegetables were more frequently preferred by girls rather than boys. A similar report was also found in another study, which reported that girls liked fruit and vegetables more than boys and boys liked fatty and sugary foods, meat, processed meat products, and eggs more than girls [25]. The known-groups validity of the dietary questionnaire was further supported by distinct patterns of association with psychological symptoms. Individuals with elevated anxiety and depression scores reported significantly lower preferences for healthy foods such as fruits and vegetables, fresh diary products, and cereals-based dish compared with those with normal psychological status. This aligns with previous research suggesting that psychological distress may reduce the appeal of healthful foods, potentially due to anhedonia or decreased motivation for meal preparation. In contrast, preferences for snacks and processed dairy products did not differ significantly across psychological symptom categories, which may reflect the palatability and rewarding properties of these energy-dense foods that remain appealing even during periods of emotional disturbance. These findings underscore the complex interplay between mental health and dietary preferences, highlighting the need for integrated approaches in nutritional interventions targeting populations with psychological symptoms [34,35].

Similar to our previous study on adolescents, BMI was not a determinant of food preference in Iranian adults [6]. The absence of significant differences in food preference scores across BMI categories suggests that body weight status may not be a determining factor in how individuals rate their liking for various foods. This finding indicates that individuals with different BMI levels share similar food preferences, and actual body weight differences likely arise from behavioral factors such as portion sizes, frequency of consumption, or energy expenditure rather than from inherent differences in what foods they prefer [36].

The convergent validity examined by the correlation between scores of psychological problems and dimensions of FPQ-adult questionnaire revealed significant negative correlations between 5 FPQ-adult subscales (ie fruit, vegetables, cereals-based dishes, animal protein, and fresh dairy products) and anxiety/depression scores. These negative correlations align closely with prior convergent-validity evidence from established food-preference instruments. For example, Wardle et al. [32] found similar negative links between HADS-D scores and preferences for dairy (*r* = −0.19) and meat/fish (*r* = −0.17) in adolescents, values almost identical to our −0.21 and −0.20 correlations. The work by Appleton et al. [37] showed that liking for complex-carbohydrate dishes inversely correlated with PHQ-9 depression scores (*r* = −0.22), matching our cereals-based dishes subscale (*r* = −0.26). Convergent evidence from epidemiological, experimental, and psychometric studies underpins our negative correlations between FPQ-adult subscales and anxiety/depression. In a Swiss survey (*n* = 20,220), 5-a-day fruit/vegetable consumers had 45% lower odds of high psychological distress (odds ratio: 0.55), and meta-analyses show that boosting fruit/vegetable intake yields moderate improvements in well-being (standardized mean difference: 0.28) and marked reductions in depressive symptoms (standardized mean difference: −0.72) via anti-inflammatory nutrients, antioxidants, and gut-microbiome effects [[38], [39], [40]]. Taken together, these convergent results reinforce that greater hedonic liking for nutrient-dense food groups consistently covaries with lower psychological distress, highlighting the convergent validity of the Persian FPQ–adult.

The wide age range of participants (19–70 y) in this study may partly explain the observed patterns in convergent validity. Our findings showed that age was positively correlated with preference for fruit (*r* = 0.120), vegetables (*r* = 0.175), cereals-based dishes (*r* = 0.092), and animal protein (*r* = 0.089), whereas negative correlations were observed for snacks (*r* = −0.162), rice and bread (*r* = −0.133), and processed dairy products and condiments (*r* = −0.136). These results align with previous research demonstrating that food preferences evolve across the lifespan. A recent study by Iizuka et al. [41] on Japanese adults found that dietary diversity and intake of beneficial foods, including fruits and dairy products, increased with age, whereas younger adults showed greater preference for convenience foods. Similarly, Moss et al. [42] reported that older adults placed greater importance on fiber content and nutritional benefits than younger adults, who were more interested in plant-based and novel food options [42]. The positive association between age and preference for nutrient-dense foods such as fruits and vegetables may reflect increased health consciousness and greater exposure to these foods over time [43]. Conversely, the negative correlation with snacks and processed products suggests that younger adults may be more receptive to hyper-palatable, convenience-oriented foods, whereas older adults develop aversions or reduce consumption due to health concerns [44]. These age-related differences in food preferences should be considered when interpreting dietary patterns and designing targeted nutritional interventions across the adult lifespan.

### Study strengths and limitations

The most important strengths of our study are its comprehensive item bank, which captures a wide spectrum of foods representative of Iranian adults and likely of populations with similar dietary habits, whereas our rigorous validation protocol, content, construct, convergent validity, internal consistency, and test–retest reliability, underpins the questionnaire’s robustness for community-based research.

Despite of its strengths, our study has several limitations. Due to the COVID-19 pandemic, about half of the questionnaires were completed electronically rather than on paper form, which may have introduced mode-related response differences. However, sensitivity analyses stratified by questionnaire format (online compared with paper) demonstrated consistent results for validity and reliability across both modes and with the total sample, suggesting that the mode of administration did not substantially influence the psychometric properties of the Persian FPQ–adult. In addition, the extensive item pool precluded the use of crossvalidation technique to further evaluate the stability of our extracted factors. Future studies with larger sample size should conduct confirmatory factor analysis to verify the factor structure we identified. Although we tried to include a broad wide variety of food items to maximize foods consumed by Iranian adults; however, we did our study in Isfahan, largest city in the center of Iran, and replication in other regions of Iran will be useful for enhancing its generalizability.

## Conclusions

The Persian version of the FPQ-adult demonstrated moderate to excellent test–retest reliability (ICCs ranging from 0.62 to 0.93) and acceptable to good internal consistency (Cronbach α ranging from 0.54 to 0.90) across all domains. The 9-factor structure extracted by EFA explained 41.73% of the total variance, supporting construct validity. Known-group validity was supported by significantly lower mean scores on the fruits, vegetables, cereals-based dishes, and higher mean animal protein dimensions among adults with elevated depression or anxiety scores than individuals with low scores. These findings indicate that the Persian FPQ–adult has adequate psychometric properties for assessing food preferences in Persian-speaking community-dwelling adults. In both epidemiological surveys and clinical research, the Persian FPQ–adult can map food-liking patterns across large samples, explore associations between taste preferences and health outcomes (e.g., cardiometabolic risk or mood disorders), and inform the design of nutrition programs that fit people’s natural choices. To deepen its validation, future studies should examine how well preference scores predict actual dietary intake and biomarkers, test responsiveness to nutrition interventions, and confirm consistency across regions, age groups, and different modes of administration. Clinically, integrating the FPQ-adult into patient assessments may help dietitians tailor meal plans to individual tastes, track shifts in food liking over time, and ultimately boost adherence and health improvements.

## Author contributions

The authors’ responsibilities were as follows—ZH: writing original draft, methodology, formal analysis, data curation, conceptualization; AF: supervision, writing – review and editing, methodology, investigation, data curation, conceptualization, funding acquisition; FH: writing – review and editing, methodology; SAT: data collection, methodology, review, and editing; and all authors: have read and approved the final version of the manuscript.

## Ethics statement

All participants were fully informed about the purpose and procedures of the study, and written informed consent was obtained prior to data collection. They were assured of the confidentiality of their responses and informed that results would be reported in aggregate form only. Ethical approval for the study was granted by the National Institute for Medical research Development (NIMAD; under grant number 982938 and approval code IR.NIMAD.REC.1398.062).

## Data availability

Data will be made available on request.

## Funding

This study was funded by the National Institute for Medical research Development (NIMAD grant number: 982938; ethical approval No. IR.NIMAD.REC.1398.062).

## Declaration of generative AI and AI-assisted technologies in the writing process

The author(s) declare that no generative AI or AI-assisted technologies were used in the writing of this manuscript.

## Conflict of interest

The authors report no conflicts of interest.
