# Supplementary material for: Psychometric Evaluation of the Persian Version of Adult Food Preferences Questionnaire (FPQ-Adult)
Source: Curr Dev Nutr. 2026 Jun 18;10(7):109399. doi: 10.1016/j.cdnut.2026.109399 (PMC13382024; doi:10.1016/j.cdnut.2026.109399)
Supplement: multimedia component 1 [file mmc1.docx]

**Table1S. Internal consistency (Cronbach's alpha) of the Persian FPQ-Adult factors by questionnaire administration format (online vs. paper).**

| Factor | Number of Items | Cronbach's Alpha (Online) n=286 | Cronbach's Alpha (Paper) n=236 | Cronbach's Alpha (Total Sample) N=522 |
| --- | --- | --- | --- | --- |
| Animal protein | 7 | 0.617 | 0.713 | 0.676 |
| Rice and bread | 8 | 0.670 | 0.692 | 0.720 |
| Cereals-based dishes | 8 | 0.696 | 0.725 | 0.683 |
| Fruit | 21 | 0.874 | 0.890 | 0.882 |
| Vegetables | 19 | 0.865 | 0.900 | 0.885 |
| Snacks | 9 | 0.797 | 0.780 | 0.788 |
| Processed dairy products and condiments | 7 | 0.704 | 0.730 | 0.717 |
| Fresh dairy products | 5 | 0.700 | 0.734 | 0.715 |
| Miscellaneous foods | 5 | 0.487 | 0.574 | 0.540 |
| Total Score | **89** | **0.902** | **0.914** | **0.900** |

**Table2S. Correlations of the FPQ-Adult subscales with age, BMI, anxiety, and depression by questionnaire administration format (online vs. paper) to assess convergent validity.**

| Sub-scale | Format | Age | BMI | Anxiety | Depression |
| --- | --- | --- | --- | --- | --- |
| Fruit | Online (n=286) | .156** | -.056 | -.184** | -.220** |
|  | Paper (n=236) | .051 | -.053 | -.069 | -.079 |
| Vegetables | Online (n=286) | .228** | -.020 | -.088 | -.151* |
|  | Paper (n=236) | .125 | -.012 | -.071 | -.076 |
| Snacks | Online (n=286) | -.191** | .009 | -.062 | -.126* |
|  | Paper (n=236) | -.124 | -.101 | .133* | .050 |
| Cereals-based dishes | Online (n=286) | -.175** | -.042 | -.056 | -.073 |
|  | Paper (n=236) | -.108 | -.137* | .073 | .070 |
| Rice and bread | Online (n=286) | .085 | -.032 | -.143* | -.224** |
|  | Paper (n=236) | .048 | -.032 | -.094 | -.105 |
| Processed dairy and condiments | Online (n=286) | -.153* | -.037 | -.020 | -.075 |
|  | Paper (n=236) | -.156* | -.067 | .016 | -.004 |
| Animal protein | Online (n=286) | .015 | -.130* | -.047 | -.117* |
|  | Paper (n=236) | .048 | .005 | -.076 | -.075 |
| Fresh dairy | Online (n=286) | -.050 | -.025 | -.092 | -.136* |
|  | Paper (n=236) | .026 | -.036 | -.046 | -.025 |
| Miscellaneous foods | Online (n=286) | -.047 | -.083 | -.101 | -.061 |
|  | Paper (n=236) | -.060 | -.006 | -.037 | -.050 |
| **Correlation is significant at the 0.01 level (2-tailed). *Correlation is significant at the 0.05 level (2-tailed). Coefficients are based on Pearson correlation coefficient or Spearman's rho rank correlation for normally and non-normally distributed variables, respectively. | | | | | |
|  |  |  |  |  |  |

**Table3S. Known-groups validity of the Persian FPQ-Adult: Comparison of subscales scores across different participant characteristics stratified by questionnaire administration format (online (n=286) vs. paper (n=236)).**

| Characteristic | Format | Fruit | Vegetables | Snacks | Cereals-based dishes | Rice and bread | Processed dairy and condiments | Animal protein | Fresh dairy | Miscellaneous |
| --- | --- | --- | --- | --- | --- | --- | --- | --- | --- | --- |
| Gender | | | | | | | | | | |
| Female(n=356) | Online | 94.05±9.36 | 82.83±10.45 | 43.61±6.59 | 35.17±3.82 | 36.94±4.68 | 25.89±5.55 | 33.49±4.73 | 20.37±3.90 | 21.07±3.93 |
|  | Paper | 96.18±9.49 | 84.30±11.56 | 44.30±7.70 | 34.36±4.41 | 37.07±5.45 | 27.37±6.08 | 32.36±5.53 | 20.82±3.80 | 19.99±4.53 |
| Male(n=166) | Online | 97.61±6.92 | 82.85±10.28 | 43.57±7.94 | 34.62±4.28 | 37.90±4.66 | 27.35±5.50 | 34.33±4.08 | 20.92±3.46 | 21.16±4.17 |
|  | Paper | 94.34±10.55 | 78.22±15.48 | 41.09±8.73 | 31.87±5.75 | 35.60±5.43 | 26.21±6.12 | 32.43±5.92 | 19.92±4.13 | 19.68±4.35 |
| p-value | Online | .002 | .987 | .962 | .291 | .124 | .046 | .166 | .267 | .855 |
|  | Paper | .170 | .001 | .004 | <.001 | .047 | .158 | .930 | .092 | .602 |
| BMI Categories | | | | | | | | | | |
| Underweight(n=12) | Online | 93.57±8.66 | 81.14±13.01 | 44.43±9.69 | 38.00±3.27 | 35.93±7.32 | 24.71±8.30 | 35.71±3.20 | 19.00±5.74 | 22.29±2.29 |
|  | Paper | 97.60±4.83 | 74.00±9.67 | 44.80±4.66 | 34.60±4.83 | 37.40±1.75 | 27.60±8.88 | 31.80±3.03 | 19.40±4.16 | 18.80±4.15 |
| Normal(n=246) | Online | 95.14±9.38 | 82.95±10.92 | 43.77±6.45 | 34.89±3.95 | 37.21±4.93 | 26.48±5.67 | 34.23±4.58 | 20.58±3.82 | 21.04±4.07 |
|  | Paper | 95.93±9.45 | 82.69±12.77 | 44.06±8.06 | 33.93±4.40 | 36.79±5.19 | 27.47±5.90 | 32.33±5.85 | 20.88±3.81 | 19.84±4.47 |
| Overweight(n=204) | Online | 95.75±8.72 | 83.35±9.87 | 43.64±7.31 | 35.27±4.09 | 37.77±4.03 | 26.42±5.45 | 33.28±4.72 | 20.63±3.46 | 21.38±3.97 |
|  | Paper | 95.14±10.28 | 82.14±14.65 | 42.44±8.62 | 33.03±5.80 | 36.46±6.00 | 26.55±6.09 | 32.79±5.09 | 20.19±4.06 | 20.13±4.38 |
| Obese(n=55) | Online | 92.81±7.45 | 81.22±9.66 | 42.76±7.40 | 34.19±3.48 | 35.77±4.87 | 25.54±5.09 | 32.86±4.04 | 20.28±4.24 | 20.22±3.98 |
|  | Paper | 95.83±8.51 | 81.11±12.56 | 41.33±6.73 | 31.39±4.98 | 35.53±5.06 | 26.11±6.53 | 32.11±5.74 | 20.50±3.61 | 19.50±4.67 |
| p-value | Online | .357 | .716 | .870 | .101 | .133 | .696 | .153 | .709 | .391 |
|  | Paper | .904 | .551 | .360 | .186 | .805 | .654 | .912 | .568 | .869 |
| Allergy |  |  |  |  |  |  |  |  |  |  |
| Yes(n=92) | Online | 91.98±10.00 | 81.04±10.88 | 45.27±6.45 | 35.21±4.15 | 36.35±5.63 | 26.48±5.89 | 33.23±4.83 | 20.35±3.96 | 21.29±3.96 |
|  | Paper | 93.70±10.39 | 79.95±15.47 | 41.57±8.59 | 31.84±5.45 | 36.28±5.57 | 25.89±6.75 | 31.86±6.48 | 19.45±4.15 | 20.57±4.03 |
| No(n=430) | Online | 95.65±8.54 | 83.19±10.27 | 43.26±7.04 | 34.98±3.92 | 37.38±4.46 | 26.26±5.51 | 33.82±4.51 | 20.55±3.75 | 21.05±4.01 |
|  | Paper | 95.93±9.75 | 82.53±12.89 | 43.45±8.11 | 33.79±4.92 | 36.58±5.45 | 27.17±5.93 | 32.51±5.45 | 20.72±3.85 | 19.71±4.54 |
| p-value | Online | .009 | .191 | .069 | .720 | .167 | .801 | .412 | .738 | .708 |
|  | Paper | .179 | .251 | .171 | .021 | .750 | .208 | .495 | .055 | .249 |
| Anxiety (HADS-A) | | | | | | | | | | |
| Normal(n=327) | Online | 96.12±8.04 | 83.61±9.92 | 43.85±7.06 | 35.15±3.92 | 37.81±4.19 | 26.53±5.57 | 34.03±4.32 | 20.76±3.62 | 21.37±3.92 |
|  | Paper | 95.81±8.78 | 82.15±13.29 | 42.18±8.46 | 33.23±4.64 | 36.85±5.30 | 26.53±5.98 | 32.83±5.18 | 20.65±3.70 | 19.91±4.51 |
| Borderline(n=102) | Online | 93.60±9.45 | 81.65±9.93 | 42.81±6.65 | 34.02±4.30 | 36.94±4.48 | 25.52±5.98 | 33.44±4.27 | 20.26±3.94 | 21.02±3.82 |
|  | Paper | 96.44±9.53 | 85.13±12.75 | 44.80±7.31 | 34.20±5.07 | 36.93±6.26 | 27.98±6.58 | 32.37±5.47 | 20.78±4.26 | 20.33±3.82 |
| Abnormal(n=80) | Online | 92.09±10.75 | 80.89±12.39 | 43.38±7.02 | 35.51±3.63 | 35.02±6.04 | 26.13±5.15 | 32.77±5.65 | 19.83±4.25 | 20.04±4.33 |
|  | Paper | 92.24±14.02 | 76.73±15.11 | 43.79±8.67 | 33.18±6.41 | 34.61±4.98 | 26.42±5.51 | 30.12±7.55 | 19.32±4.49 | 18.64±4.84 |
| p-value | Online | .009 | .190 | .636 | .136 | .001 | .522 | .210 | .282 | .122 |
|  | Paper | .125 | .020 | .123 | .458 | .095 | .300 | .049 | .187 | .207 |
| Depression (HADS-D) | | | | | | | | | | |
| Normal(n=340) | Online | 96.34±7.72 | 83.74±9.78 | 44.25±7.05 | 35.22±3.97 | 38.09±4.14 | 26.62±5.63 | 34.23±4.31 | 20.97±3.61 | 21.38±3.81 |
|  | Paper | 96.37±8.31 | 82.47±13.18 | 42.79±8.09 | 33.15±4.76 | 36.91±5.28 | 26.82±6.30 | 32.66±5.06 | 20.61±3.89 | 19.88±4.37 |
| Borderline(n=102) | Online | 93.57±9.53 | 82.09±10.53 | 41.67±6.63 | 34.19±3.92 | 36.15±4.88 | 25.47±5.77 | 33.19±4.17 | 19.37±3.82 | 20.21±3.84 |
|  | Paper | 95.16±12.04 | 82.23±13.02 | 43.05±9.07 | 34.00±5.76 | 36.08±6.00 | 27.32±5.62 | 32.16±6.08 | 19.81±3.89 | 19.48±4.34 |
| Abnormal(n=61) | Online | 90.87±11.47 | 79.56±12.41 | 43.31±6.68 | 35.31±3.86 | 34.53±5.56 | 25.95±4.91 | 32.05±5.80 | 20.04±4.23 | 21.05±4.89 |
|  | Paper | 90.45±13.71 | 79.68±12.41 | 43.59±6.49 | 33.45±5.39 | 35.05±5.63 | 27.00±5.38 | 31.77±6.60 | 20.55±3.61 | 18.82±4.91 |
| p-value | Online | .001 | .061 | .045 | .199 | <.001 | .355 | .015 | .012 | .149 |
|  | Paper | .031 | .646 | .907 | .616 | .268 | .891 | .712 | .477 | .539 |
| Data are presented as mean ± standard deviation. P-values were derived from independent t-test (for binary characteristics) and one-way ANOVA (for characteristics with more than two categories), as appropriate, comparing subscale scores between categories of each characteristic within each questionnaire administration format (online vs. paper). The discrepancy between the number of participants across categories of some variables and the total sample size is due to missing data in those variables. | | | | | | | | | | |
